# Cognitive decline and risk of stroke in older women: a cohort study

**DOI:** 10.1186/s12877-026-07816-w

**Published:** 2026-06-16

**Authors:** Ricarda S. Schulz, Toivo Glatz, Julie E. Buring, Jae H. Kang, Tobias Kurth, Pamela M. Rist

**Affiliations:** 1 Institute of Public Health, Charité – Universitätsmedizin Berlin, Corporate member of Freie Universität Berlin and Humboldt-Universität zu Berlin, Berlin, Germany; 2 Division of Preventive Medicine, Department of Medicine, Brigham and Women’s Hospital, Harvard Medical School, Boston, MA USA; 3 Channing Division of Network Medicine, Department of Medicine, Brigham and Women’s Hospital, Harvard Medical School, Boston, MA USA

**Keywords:** Cognition, Cognitive decline, Stroke, Women, Cohort study, Epidemiology

## Abstract

**Background:**

Impaired cognitive function has been linked to an elevated risk of stroke. However, the effect of cognitive change, conceptualized as a dynamic process, has received limited attention. We therefore aimed to estimate the effect of cognitive decline on the risk of stroke in older women.

**Methods:**

Our analyses were based on data from the cognitive cohort of the Women’s Health Study composed of 6,377 US female health professionals aged 65 years and older at baseline. We included women without a history of stroke who had complete information on global cognitive performance change. Women were categorized into quintiles based on their 4-year global cognitive performance change and followed for self-reported incident stroke confirmed by a physician Endpoints Committee through December 31, 2022. We used multivariable-adjusted Cox proportional hazards regression models to estimate hazard ratios (HRs) and corresponding 95% confidence intervals (CIs) of the association between cognitive change and stroke. Secondary analyses assessed the risk for stroke subtypes, lowest 10% and 20% of the cognitive change distribution, and changes in verbal memory.

**Results:**

5,093 women with a mean age of 66 years (SD = 3.93) were analyzed. The mean follow-up time was 11.6 years per person during which 302 incident stroke cases were reported. Compared to the 5th quintile (greatest improvement), the adjusted HR for stroke was 0.76 (95%CI [0.53–1.10]) for the 4th, 0.91 (95%CI [0.64–1.29]) for the 3rd, 0.95 (95%CI [0.67–1.34]) for the 2nd, and 1.02 (95%CI [0.72–1.45]) for the 1st quintile characterized by the greatest decline. Similar patterns were observed in secondary analyses. Women in the lowest 10% of global cognitive change exhibited a non-significant increase in the risk of stroke compared to the rest of the distribution (HR_adj_ = 1.25 (95%CI [0.86–1.82]).

**Conclusions:**

Large declines in cognitive performance were not strongly associated with stroke risk in this cohort of older female health professionals. However, future studies should further explore whether cognitive trajectories may influence risk of stroke and assess the generalizability of our findings, particularly to men.

**Supplementary Information:**

The online version contains supplementary material available at 10.1186/s12877-026-07816-w.

## Introduction

Over the next decades, the prevalence of individuals experiencing cognitive decline or cognitive impairment, as well as pathological cognitive decline as observed in patients affected by conditions such as Alzheimer’s Disease [1], Parkinson’s Disease [2], or frontotemporal dementia [3] is expected to increase, due to demographic aging [4].

In addition to the burden on affected individuals and the societal burden in terms of financial and personnel resources, a previous meta-analysis suggested that cognitive impairment also increases the risk of subsequent stroke by nearly 40% [5]. Another study reported that participants characterized by low executive function had an approximately 50% increased risk of incident stroke, while lower memory function was not associated with an increase in stroke risk [6]. The occurrence of stroke events among those with cognitive impairment further increases the burden on both the individual and society given the burden of post-stroke disability and the substantial costs associated with the acute treatment of stroke and post-stroke care [7–10].

Pathophysiological mechanisms that are hypothesized to increase the risk for stroke in patients affected by impairment in cognitive function, include the presence of white-matter hyperintensities [11, 12] silent brain infarctions [13, 14], and cerebral microbleeds [15], as well as disturbances of cerebrovascular hemodynamics [16]. These radiological markers are considered manifestations of cerebral small vessel disease and are associated with increased risks of both ischemic and hemorrhagic stroke, potentially through distinct underlying injury pathways [17–20].

While prior work has shown that cognitive impairment measured at a single time point - particularly when using a clinical cut-off score (e.g., Mini-Mental State Examination (MMSE) < 24) - is associated with future stroke risk [5], less is known about how changes in cognitive function over time influence this risk. Most studies to date have focused on advanced stages of cognitive decline, operationalized as clinically defined cognitive impairment, rather than on longitudinal trajectories of cognitive performance in relation to stroke risk [5].

Thus, less focus was set on individuals who initially exhibited high cognitive performance at baseline and remained on a relatively high-functioning trajectory over time, and would not be classified as “cognitive impaired” in a follow-up assessment, despite having experienced a measurable decline in cognitive function across repeated measures. Additionally, many studies have relied solely on MMSE scores to assess cognitive function at a single time point, and few studies have examined individual cognitive domains [6, 11, 16]. Finally, studies were often unable to examine ischemic and hemorrhagic stroke separately [5].

The primary aim of this study, therefore, was to estimate the effect of cognitive decline on the risk of subsequent total stroke among women aged 65 years and older in the cognitive cohort of the Women’s Health Study. As secondary analyses we examined this potential association for stroke subtypes (ischemic and hemorrhagic).

## Methods

### Study population

The Women’s Health Study (WHS) (ClinicalTrials.gov Identifier: NCT00000479; study registration date: first posted (estimated) October 28th, 1999) was a large, randomized, placebo-controlled trial evaluating in a 2 × 2 factorial design the effects of low-dose aspirin and vitamin E in the primary prevention of cardiovascular disease (CVD) and cancer in women. In-depth descriptions of the design, methodology, and main results have been previously published [21, 22]. In brief, at study baseline (1992–1995), 39,876 US female health professionals, at least 45 years of age and free of baseline history of CVD, cancer (except nonmelanoma skin cancer), and other major diseases, were recruited and randomized into the trial [23]. After the end of the main trial on March 31st, 2004, an observational follow-up of women consenting to continue their participation was initiated and is currently ongoing.

All women provided written informed consent for participation in the WHS, which was approved by the Institutional Review Board of the Brigham and Women’s Hospital, Boston, MA (IRB number observational follow-up: 2004P001661).

### Population for analysis and follow-up

Within the WHS, a cognitive cohort was established in 1998 (IRB number cognitive cohort: 1999P002903). To be eligible, women had to be at least 65 years old and be active participants of the WHS (*N* = 7,175). A first cognitive performance assessment by telephone was completed by 6,377 women (88.9% of women fulfilling the eligibility criteria for inclusion). The women underwent subsequent cognitive follow-up assessments and for the present analyses, we included women with complete information on change in global cognitive performance between the first and last cognitive interview, which was about four years after the first assessment [24]. Detailed information on the study workflow is provided in a timeline plot in the supplementary material (eFigure1). Women with a stroke event before the last cognitive assessment and those who did not participate in the follow-up cognitive interviews were excluded. Furthermore, we excluded women with missing cognitive test data in any of the tests at either the first or the last assessment required to compute global cognitive performance change (eFigure2 - flow chart for participant selection). Outcome follow-up was administratively censored at the end of the observation period, December 31, 2022.

### Assessment of cognitive performance

Cognitive performance was assessed by a cognitive test battery made up of five separate tests carried out by trained nurses [24]. General cognition was administered by the Telephone Interview of Cognitive Status (TICS), a telephone adaptation of the MMSE [25] with scores ranging from 0 to 41 points. This test has been proven to have high validity and reliability in measuring global cognitive functioning [26]. Verbal memory was assessed using the East Boston Memory Test, which includes immediate and delayed recall of a short paragraph containing 12 key elements. Participants were asked to repeat the elements immediately after hearing the paragraph and again after 15 min, with scores ranging from 0 to 12 [27]. As an additional measure of delayed verbal memory, the TICS 10-word list was also assessed, with a score range of 0 to 10 points. Finally, to test category fluency, such as in language and executive functioning, women were asked to name as many animals as possible in one minute [27, 28].

To define the exposure, i.e., global cognitive performance change, global test scores were generated by averaging the *z*-scores of the baseline performance in each test, a method previously applied in the context of the WHS [27]. In more detail, cognitive test scores at each time point were standardized (*z*-scores) using the baseline distribution (mean and standard deviation). The same standardization parameters were applied to follow-up scores to ensure comparability over time. To categorize women according to cognitive performance change, we computed the quintiles of the global *z*-score change between the first and last cognitive assessment four years later.

### Assessment and classification of incident stroke

After enrollment in the main trial, women were sent follow-up questionnaires at six and twelve months, and annually thereafter, which gathered information on demographics, lifestyle, and health-related details, including physician-diagnosed stroke events. Medical records were requested for self-reported events, and a physician-based Endpoints Committee confirmed or disconfirmed all self-reported events. A stroke event was defined as the occurrence of a focal neurological deficit resulting from vascular mechanisms that lasted at least 24 h, thereby excluding transient ischemic attacks. For identifying stroke subtypes (e.g., ischemic versus hemorrhagic), the modified Trial of Org 10172 in Acute Stroke Treatment (TOAST) criteria [29] were applied to clinical information or imaging results. A high interobserver agreement for classifying major stroke subtypes has been demonstrated for the WHS [30].

### Statistical analysis

#### Descriptive analysis of the study cohort

We calculated mean values and standard deviations (SD) for continuous data and proportions of frequencies for categorical variables for baseline demographic and clinical characteristics according to global cognitive performance change quintiles. Additionally, we compared the baseline characteristics and test scores of women who did not participate in the last cognitive assessment (lost to follow-up) with those of the women who completed the last assessment to assess possible bias due to differential loss to follow-up. Finally, we comparatively describe the mean test score for each test at each of the two assessment points considered in the analysis across the cognitive change quintiles.

#### Primary analysis

We calculated person-time from the completion of the last cognitive interview until the occurrence of the first stroke, loss to follow-up, death, or the end of follow-up (administrative censoring set to December 31, 2022), whichever occurred first.

Kaplan-Meier curves were visually evaluated to identify potential violations of the proportional hazard assumption. Additionally, we formally assessed the former using Schoenfeld residuals. We further performed log-rank tests to test for differences between the survival curves of the groups compared.

To estimate unadjusted and adjusted hazard ratios (HRs) and corresponding 95%CIs for the effect of global cognitive performance change on the risk of stroke, Cox proportional hazards regression models [31] were applied, using the 5th quintile with the greatest improvement as the reference group.

We adjusted our models for factors that could potentially confound the relationship between cognitive trajectory and stroke. Variable selection for the adjustment set was based on expert knowledge and a review of the literature in the field. To clarify our adjustment approach, we constructed a Directed Acyclic Graph (DAG) using the software DAGitty [32] (eFigure3). The multivariable models were adjusted for the following baseline variables: age (years), highest attained education (Licensed Practical Nurse/Licensed Vocational Nurse, 2-yr Assoc./Registered Nurse, 3-yr Assoc./Registered Nurse, Bachelor, Master, Doctorate/MD), body mass index (BMI) computed as kg/m², strenuous physical activity (rarely/never, < 1 time/week, 1 time/week, 2–3 times/week, 4–6 times/week, 7 + times/week), smoking status (never, current, past), alcohol use (rarely/never, 1–3 drinks/month, 1–6 drinks/week, 1 + drinks/day), and the following information on medical history recorded as binary (i.e., yes or no): self-reported diabetes, hypertension (defined as a medical doctor diagnosis of systolic blood pressure ≥ 140 or diastolic blood pressure ≥ 90 at baseline), treatment for high blood pressure, high cholesterol (based on medical doctor diagnosis at enrollment, self-report cholesterol 240+, or use of cholesterol lowering medication), and treatment for high cholesterol, as well as for use of hormone replacement therapy (never, current, past). In general, validation studies in the WHS have shown that study participants reliably report the presence or absence of health conditions [33–35].

We further assessed a potential linear dose-response association between cognitive change and stroke risk by additionally adding a median-based trend variable to the primary Cox proportional hazards regression model.

To mitigate potential selection bias due to non-participation in the last cognitive assessment, stabilized inverse probability of treatment weights for participation were estimated. These weights were subsequently applied in a sensitivity analysis of the primary model to account for bias due to differential attrition.

#### Secondary analyses

We dichotomized our exposure and applied the survival models specified in our primary analysis to compare the risk of stroke among the bottom 20% and then the bottom 10% of the cognitive change distribution with the rest of the study population.

The risk of stroke was also assessed according to major stroke subtypes, i.e., ischemic and hemorrhagic stroke. For ischemic stroke, we analyzed the data in the quintiles. Due to low case numbers for hemorrhagic stroke (*N* = 34), we only present counts by exposure group descriptively and refrained from formal statistical analysis.

Third, as verbal memory is one of the strongest predictors for Alzheimer’s Disease (AD), the leading cause of dementia [36], and as AD has been linked with increased risk of stroke [37], we repeated the primary analysis using verbal memory change quintiles between the first and last cognitive assessments as the exposure [38]. For the computation of the verbal memory change quintiles, immediate and delayed recalls of the East Boston Memory Test and the delayed recall of TICS 10 word-list were considered. This procedure has been previously applied in the context of the WHS cognitive cohort [27].

For all secondary analyses, we applied Cox proportional hazards regression models and the same adjustment set as for our primary analysis. Unless otherwise specified above, the 5th quintile (greatest improvement) was set as the reference.

### Handling of missing data

Missingness across all variables included in the multivariable models was low (< 1.6% of the study population). Additional information on missing data is provided in the supplement (eFigure4). For BMI, the mean value was imputed. For categorical variables, the most frequent category was assigned: ‘*rarely/never*’ for alcohol use and physical activity, ‘*3-yr Assoc./RN*’ for education, ‘*never*’ for baseline use of hormone replacement therapy, and *“no”* for baseline history of diabetes, hypertension, treatment of high blood pressure, high cholesterol, and treatment for high cholesterol. For the smoking status the *‘past user’* category was imputed.

*p*-values ≤ 0.05 were considered statistically significant under the assumption of a type I error of 5% in two-tailed tests. The statistical analyses were performed using R [39] (version 4.4.2) and RStudio (version 2024.09.1).

## Results

Of the 6,337 women who participated in the first cognitive assessment, 1,151 women (18.2%) did not participate in the last cognitive assessment four years later. Non-participants at baseline were more likely to be current smokers (13.9% vs. 9.1%), to rarely or never drink alcohol (52.8% vs. 46.5%) or rarely or never engage in strenuous physical activity (46.1% vs. 42.5%). Also, they were more likely to have a history of diabetes (5.0% vs. 3.2%), hypertension (43.6% vs. 39.3%) or to receive treatment for high blood pressure (25.3% vs. 21.7%) than those who were included in this study (see Table A1).

After excluding women without last cognitive assessment (*n* = 1,151), missing information on test performance in one of the cognitive tests required for global cognitive change computation at first and last interview (*n* = 12), and those with a stroke prior to this assessment (*n* = 122), we included 5,093 women in our analysis and categorized these women into quintiles based on their global cognitive performance change. Overall, the baseline characteristics were similar across the quintiles for both demographic and clinical data (see Table 1). The women in the study had an overall mean age of 66 years (SD: 3.9; median [min, max]: 65.3 [60.4, 87.1] years) at baseline. At the first cognitive assessment, their mean age was 72 years (SD: 3.92), with a median [min; max] of 71 [66; 93] years.

**Table 1:** Baseline characteristics of included women in the WHS cognitive cohort by global cognitive performance quintiles

	1^st^ quintile (*n* = 1,019)	2^nd^ quintile (*n* = 1,018)	3^rd^ quintile (*n* = 1,019)	4^th^ quintile (*n* = 1,018)	5^th^ quintile (*n* = 1,019)	Overall (*N* = 5,093)
Age						
Mean (SD)	67.0 (4.31)	66.1 (4.00)	65.8 (3.84)	65.8 (3.75)	65.6 (3.57)	66.0 (3.93)
Median [Min, Max]	66.4 [60.5, 83.1]	65.3 [60.5, 87.1]	64.9 [60.4, 82.8]	65.1 [60.5, 82.3]	64.8 [60.4, 80.2]	65.3 [60.4, 87.1]
BMI						
Mean (SD)	25.8 (4.36)	25.9 (4.58)	25.8 (4.49)	25.9 (4.32)	25.7 (4.41)	25.8 (4.44)
Median [Min, Max]	25.0 [17.2, 47.5]	25.3 [15.7, 48.0]	25.0 [16.0, 45.3]	25.1 [17.7, 51.5]	25.0 [16.1, 48.4]	25.1 [15.7, 51.5]
Missing	1 (0.1%)	0 (0%)	0 (0%)	0 (0%)	0 (0%)	1 (0.0%)
Highest attained education						
LPN/LVN, associate’s degree, registered nurse	662 (65.0%)	641 (63.0%)	683 (67.0%)	657 (64.5%)	678 (66.5%)	3,321 (65.2%)
Bachelor’s degree or higher education	346 (34.0%)	362 (35.6%)	323 (31.7%)	338 (33.2%)	323 (31.7%)	1,692 (33.2%)
Missing	11 (1.1%)	15 (1.5%)	13 (1.3%)	23 (2.3%)	18 (1.8%)	80 (1.6%)
Smoking status						
Never	536 (52.6%)	537 (52.8%)	541 (53.1%)	541 (53.1%)	542 (53.2%)	2,697 (53.0%)
Past	388 (38.1%)	387 (38.0%)	383 (37.6%)	394 (38.7%)	380 (37.3%)	1,932 (37.9%)
Current	93 (9.1%)	94 (9.2%)	94 (9.2%)	82 (8.1%)	96 (9.4%)	459 (9.0%)
Missing	2 (0.2%)	0 (0%)	1 (0.1%)	1 (0.1%)	1 (0.1%)	5 (0.1%)
Strenuous physical activity						
Rarely/never	427 (41.9%)	442 (43.4%)	402 (39.5%)	442 (43.4%)	437 (42.9%)	2,150 (42.2%)
<1 time/week	157 (15.4%)	188 (18.5%)	176 (17.3%)	167 (16.4%)	152 (14.9%)	840 (16.5%)
1 time per week	93 (9.1%)	73 (7.2%)	87 (8.5%)	76 (7.5%)	94 (9.2%)	423 (8.3%)
2–3 times/week	204 (20.0%)	202 (19.8%)	241 (23.7%)	211 (20.7%)	215 (21.1%)	1,073 (21.1%)
≥ 4 times/week	136 (13.3%)	112 (11.0%)	111 (10.9%)	122 (12.0%)	121 (11.9%)	602 (11.8%)
Missing	2 (0.2%)	1 (0.1%)	2 (0.2%)	0 (0%)	0 (0%)	5 (0.1%)
Alcohol use						
Rarely/never	481 (47.2%)	481 (47.2%)	467 (45.8%)	474 (46.6%)	467 (45.8%)	2,370 (46.5%)
1–3 drinks/month	124 (12.2%)	112 (11.0%)	113 (11.1%)	112 (11.0%)	145 (14.2%)	606 (11.9%)
1–6 drinks/week	294 (28.9%)	284 (27.9%)	308 (30.2%)	309 (30.4%)	296 (29.0%)	1,491 (29.3%)
1 + drinks/day	120 (11.8%)	141 (13.9%)	129 (12.7%)	121 (11.9%)	111 (10.9%)	622 (12.2%)
Missing	0 (0%)	0 (0%)	2 (0.2%)	2 (0.2%)	0 (0%)	4 (0.1%)
Hormone replacement therapy use						
Never	443 (43.5%)	410 (40.3%)	408 (40.0%)	468 (46.0%)	433 (42.5%)	2,162 (42.5%)
Past	178 (17.5%)	200 (19.6%)	190 (18.6%)	159 (15.6%)	175 (17.2%)	902 (17.7%)
Current	397 (39.0%)	407 (40.0%)	420 (41.2%)	390 (38.3%)	409 (40.1%)	2,023 (39.7%)
Missing	1 (0.1%)	1 (0.1%)	1 (0.1%)	1 (0.1%)	2 (0.2%)	6 (0.1%)
Baseline history of diabetes						
No	983 (96.5%)	980 (96.3%)	989 (97.1%)	986 (96.9%)	995 (97.6%)	4,933 (96.9%)
Yes	36 (3.5%)	37 (3.6%)	30 (2.9%)	32 (3.1%)	24 (2.4%)	159 (3.1%)
Missing	0 (0%)	1 (0.1%)	0 (0%)	0 (0%)	0 (0%)	1 (0.0%)
Baseline history of hypertension						
No	622 (61.0%)	617 (60.6%)	630 (61.8%)	617 (60.6%)	625 (61.3%)	3,111 (61.1%)
Yes	397 (39.0%)	400 (39.3%)	389 (38.2%)	400 (39.3%)	394 (38.7%)	1,980 (38.9%)
Missing	0 (0%)	1 (0.1%)	0 (0%)	1 (0.1%)	0 (0%)	2 (0.0%)
Baseline treatment of high blood pressure						
No	801 (78.6%)	795 (78.1%)	803 (78.8%)	792 (77.8%)	806 (79.1%)	3,997 (78.5%)
Yes	217 (21.3%)	223 (21.9%)	215 (21.1%)	226 (22.2%)	212 (20.8%)	1,093 (21.5%)
Missing	1 (0.1%)	0 (0%)	1 (0.1%)	0 (0%)	1 (0.1%)	3 (0.1%)
Baseline history of hypercholesterolemia (cholesterol 240+)						
No	567 (55.6%)	578 (56.8%)	582 (57.1%)	570 (56.0%)	590 (57.9%)	2,887 (56.7%)
Yes	451 (44.3%)	439 (43.1%)	437 (42.9%)	447 (43.9%)	429 (42.1%)	2,203 (43.3%)
Missing	1 (0.1%)	1 (0.1%)	0 (0%)	1 (0.1%)	0 (0%)	3 (0.1%)
Baseline treatment of hypercholesterolemia						
No	956 (93.8%)	943 (92.6%)	957 (93.9%)	958 (94.1%)	957 (93.9%)	4,771 (93.7%)
Yes	62 (6.1%)	75 (7.4%)	60 (5.9%)	58 (5.7%)	60 (5.9%)	315 (6.2%)
*z*-Score of global cognition at first assessment						
Mean (SD)	0.249 (0.610)	0.208 (0.573)	0.0904 (0.562)	0.0198 (0.551)	-0.299 (0.556)	0.0536 (0.603)

*BMI* Body Mass Index (calculated as weight in kilograms divided by height in meters squared), *LPN/LVN* Licensed Practical Nurse/Licensed Vocational Nurse; global cognitive performance change thresholds: 1st quintile: (-4.687, -0.512), 2nd quintile: (-0.512, -0.119), 3rd quintile: (-0.119, 0.188), 4th quintile: (0.188, 0.568), 5th quintile: (0.568, 2.762)

Among them, 53.0% reported never being a smoker, and 46.5% indicated that they rarely or never drink alcohol. Regarding their physical activity, 42.2% stated that their strenuous physical activity level was *‘rarely/never’*. A history of hypertension was documented by 38.9% of the women and 43.3% reported a history of high cholesterol at baseline.

Table A2 displays information on women’s test scores for each of the cognitive tests performed at the first and the last assessment by cognitive change quintile assignment. The global cognitive performance change thresholds for the quintile assignment were the following: 1st quintile: (-4.687, -0.512), 2nd quintile: (-0.512, -0.119), 3rd quintile: (-0.119, 0.188), 4th quintile: (0.188, 0.568), 5th quintile: (0.568, 2.762). Notably, individuals in the 1st and 2nd quintiles experienced declines, whereas those in the 4th and 5th quintiles showed improved cognitive performance over time.

Overall, the women who did not take part in the last cognitive assessment performed slightly worse on average over all cognitive tests in the first assessment than women who also completed the last assessment (see Table A3).

### Primary analysis

During a mean follow-up time of 11.6 years per person (median: 12 years; interquartile range: 6.7–17.2 years per person; sum across participating women: 58,991.2 person-years), 302 (5.9%) confirmed incident stroke events were recorded of which 60 (19.9%) cases were observed among the 1st quintile (i.e., those who showed the largest cognitive decline) and 67 (22.2%) cases were observed among the 5th quintile (i.e., those who improved the most; reference group).

The visual inspection of the Kaplan-Meier did not indicate violations of the proportional hazards assumption (eFigure5), which was further supported by formal testing using Schoenfeld residuals (global Schoenfeld test *p* = 0.45). Performing log-rank tests did not reveal an overall shift in probability of stroke between the groups (*p*-value ≥ 0.1).

**Fig. 1:**
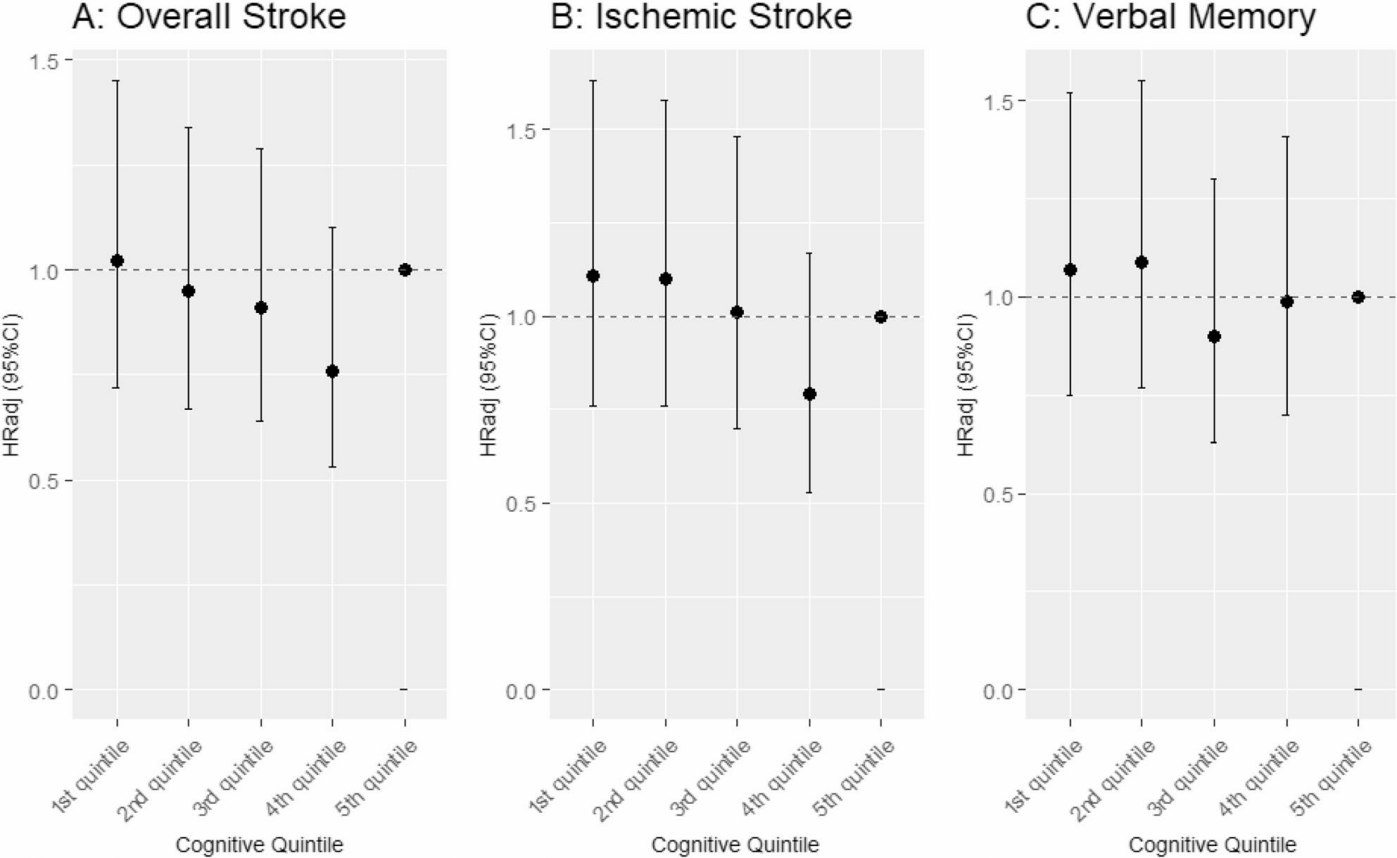
Adjusted HRs for incident stroke risk and ischemic stroke (global cognition) and by verbal memory. Global cognitive change quintiles and **A**: risk for any incident stroke (primary analysis) and **B**: risk for incident ischemic stroke; **C**: Verbal memory change quintiles and risk for any incident stroke

In Table 2, we report the HRs and corresponding 95%CIs obtained by unadjusted and multivariable adjusted Cox proportional hazards regression model analyses. Overall, we did not find statistically significant associations between cognitive performance change and the risk of stroke or stroke subtypes. Using the 5th quintile (those with the greatest improvement) as the reference, the adjusted HR (HR_adj_) for stroke was 0.76 (95%CI [0.53–1.10]) for the 4th quintile, slightly increasing to 0.91 (95%CI [0.64–1.29]) for the 3rd quintile. The HR_adj_ then increased to 0.95 (95%CI [0.67–1.34]) for the 2nd and further to 1.02 (95%CI [0.72–1.45]) for the 1st quintile with the greatest decline in cognitive function (Fig. 1, Panel A).

**Table 2:** HRs for total and ischemic stroke by global cognition and for stroke by verbal memory

Global cognitive performance quintiles:	1^st^ quintile (*n* = 1,019) HR (95%CIs)	2^nd^ quintile (*n* = 1,018) HR (95%CIs)	3^rd^ quintile (*n* = 1,019) HR (95%CIs)	4^th^ quintile (*n* = 1,018) HR (95%CIs)	5^th^ quintile (*n* = 1,019) HR [reference]
Stroke, *N*	60	62	60	53	67
unadjusted	1.09 (0.77–1.55)	0.98 (0.69–1.38)	0.92 (0.65–1.30)	0.77 (0.53–1.10)	1 (ref)
adjusted^1^	1.02 (0.72–1.45)	0.95 (0.67–1.34)	0.91 (0.64–1.29)	0.76 (0.53–1.10)	1 (ref)
	(*n* = 1,013)	(*n* = 1,015)	(*n* = 1,014)	(*n* = 1,010)	(*n* = 1,007)
Ischemic stroke, *N*	54	59	55	45	55
unadjusted	1.19 (0.82–1.74)	1.12 (0.78–1.62)	1.02 (0.70–1.48)	0.79 (0.53–1.17)	1 (ref)
adjusted^1^	1.11 (0.76–1.63)	1.10 (0.76–1.58)	1.01 (0.70–1.48)	0.79 (0.53–1.17)	1 (ref)
Verbal memory performance quintiles:	(*n* = 1,027)	(*n* = 1,014)	(*n* = 1,038)	(*n* = 995)	(*n* = 1,019)
Stroke, *N*	61	63	55	61	62
unadjusted	1.12 (0.78–1.59)	1.08 (0.76–1.53)	0.89 (0.62–1.28)	0.97 (0.68–1.38)	1 (ref)
adjusted^1^	1.07 (0.75–1.52)	1.09 (0.77–1.55)	0.90 (0.63–1.30)	0.99 (0.70–1.41)	1 (ref)

*Abbreviations*: *CI* Confidence Interval, *HR* Hazard Ratio, *ref* reference group

^1^Adjusted for age, strenuous physical activity, alcohol use, smoking status, BMI (calculated as weight in kilograms divided by height in meters squared), highest attained educational level, hormone replacement therapy, high blood pressure, treatment of high blood pressure, high cholesterol, treatment of high cholesterol, and diabetes at baseline

Using a median-based trend variable, no evidence of a linear dose-response association between cognitive performance and stroke risk was observed (HR: 0.98 [95%CI (0.90–1.06)]; *p* = 0.57).

The weighted results of the sensitivity analysis were similar to the primary analysis (Table A4), suggesting that selection bias due to differential participation is unlikely to have substantially affected the findings.

### Secondary analyses

Comparing the 1st quintile to the rest of the global cognitive change distribution, the obtained HR_adj_ for stroke was 1.13 (95%CI [0.85–1.50]), indicating a small, non-significant increase in risk of stroke for women with the largest decline in cognitive performance. Similarly, those in the bottom 10% of the cognitive change distribution (*N* = 510; cut-off = -0.821) experienced 31 stroke events and also had a small, non-significant increase in the risk of stroke when compared to the rest of the distribution of change scores (HR_adj_ = 1.25 (95%CI [0.86–1.82])).

Analyzing the risk for stroke by stroke subtypes, for ischemic stroke (*n* = 268), we observed a similar pattern of HRs across cognitive quintiles as for stroke in general, with no significant associations seen (Table 2; Fig. 1, Panel B).

For hemorrhagic stroke (*n* = 34), 12 stroke events were recorded for the 5th quintile (i.e., the greatest improvement over time), 8 for the 4th, 5 for the 3rd, 3 for the 2nd quintile, and 6 for the 1st quintile (i.e., those with the greatest declines).

The verbal memory change thresholds for the quintile assignment were the following: 1st quintile: (-5.002, -0.627), 2nd quintile: (-0.627, -0.15), 3rd quintile: (-0.15, 0.264), 4th quintile: (0.264, 0.763), 5th quintile: (0.763, 3.326). Compared to the 5th quintile, we observed no increases in the risk of stroke for the other four quartiles (Table 2; Fig. 1, Panel C).

## Discussion

In this large prospective cohort of older women, no strong associations between cognitive performance declines and stroke risk were observed. The same pattern was seen when analyzing ischemic stroke as the outcome (Fig. 1, Panel A and B). When analyzing the stroke risk for lowest 20% and 10% compared to the rest of the global cognitive performance distribution, we observed small, non-significant increases in the risk of stroke.

### Comparison with other studies

Most prior studies have dichotomized the exposure as either cognitively impaired or not, thereby focusing on advanced stages of cognitive decline. For example, in a meta-analysis focusing on cognitive impairment and risk of future stroke, baseline cognitive impairment was found to be associated with an increased risk of stroke, RR = 1.39 (95%CI [1.24–1.56]). When the analysis was restricted to studies that used a widely adopted definition of cognitive impairment, specifically, a MMSE score below 25 or its closest equivalent, the estimated risk was even higher RR = 1.64 (95%CI [1.46–1.84]) [5].

In contrast to an approach based on a single MMSE cutoff value the Framingham Offspring cohort used a comprehensive cognitive test battery among 1,679 participants aged 55 years or older (mean age 65.7 ± 7.0) free of dementia, stroke, and other neurological conditions that could affect cognition. Participants scoring more than 1.5 SDs below the age- and education-adjusted mean on an executive function test had an increased risk of future stroke (HR = 2.27 (95%CI [1.06–4.85])) [16].

In addition to the studies on general cognition or executive function, results from the Health and Retirement Study cohort (*N* = 19,087) suggest that memory impairment, defined as a combined immediate and delayed recall score of fewer than 6 out of 20 possible points on a 10-word list recall task, was associated with a higher risk of incident stroke (HR = 1.26 (95%CI [1.13–1.41]) [11].

A further study examined 3,926 older adults participating in the Prospective Study of Pravastatin in the Elderly at Risk (PROSPER) with a mean age of 75 years with increased cardiovascular risk or a history of CVD events but free of dementia at baseline (defined as MMSE ≥ 24). The study focused on specific cognitive domains, with executive function assessed by *z*-standardized scores on the Stroop Color-Word Test and the Letter Digit Substitution Test, and memory evaluated through immediate and delayed recall in the Picture Learning Test. Over a median follow-up of 3.2 years, participants in the lowest tertile of executive function showed an increased risk of incident stroke (HR = 1.51 (95%CI [0.99–2.30])) although this increase in risk was not statistically significant. Conversely, lower memory performance was not associated with increase in stroke risk (HR = 0.87 (95%CI [0.57–1.32])) [6].

In contrast to most studies, the Atherosclerosis Risk in Communities (ARIC) cohort observed no association between cognitive function and the incidence of stroke [40].

Risk factors for stroke, as well as cognitive impairment, are not inevitably equally distributed across age groups, and it is possible that results might differ for different study populations with diverging age and risk factor profiles. Specifically, cognitive impairment has been suggested as a potentially distinct risk factor for stroke among older adults [41]. The ARIC cohort was slightly younger (48–67 years) than prior cohort studies, which may partially explain the lack of association.

However, the approach of dichotomizing individuals as cognitive impaired or not overlooks individuals who, despite experiencing measurable decline, may have initially had a high level of cognitive functioning and thus were not classified as being “cognitively impaired” on follow-up assessments. Additionally, most studies have focused on a cognitive performance measure at one point in time and did not address how changes in cognitive function over time, i.e., cognitive decline, influence stroke risk.

A few studies have examined cognitive decline over time, employing a design similar to the current study. Among the 30,959 participants with a history of CVD or high-risk diabetes enrolled in the ONTARGET and TRANSCEND trials, a decline of two or more points in MMSE score, assessed between baseline, at the 2-year follow-up and the penultimate study visit, was associated with an increased risk of stroke (HR: 1.34 (95%CI [1.11–1.61]) [42]. In contrast to these findings from a cohort at high CVD risk, including individuals with established CVD, the WHS only enrolled women free of CVD at baseline. This difference in baseline risk profile may partly contribute to the stronger association between cognitive decline and stroke observed in the analysis of the two trials. Furthermore, while the trials assessed cognitive function using a single measure i.e., the MMSE, the WHS cognitive cohort employed a neuropsychological test battery capturing a broader range of cognitive domains. These methodological and population differences should be considered when interpreting the cross-study comparisons and assessing the generalizability of the findings.

### Potential mechanisms and pathophysiology

Previous research suggests that cognitive impairment shares common pathophysiological mechanisms with stroke, and that impairment of cognitive performance correlates with indicators of subclinical cerebrovascular pathology [5, 11]. For example, compared to those without cognitive impairment, individuals with cognitive impairment have more silent brain infarcts [13, 14], white-matter hyperintensities [11, 12], microbleeds, abnormalities of hemodynamics of the cerebrovascular system [43], and amyloid deposition in cerebral vessels [44], which themselves may contribute to stroke risk [5].

Given these pathophysiological commonalities, structural brain imaging measures as well as cognitive tests have been used to predict stroke risk. Participants who experienced either executive function deficits, lower total brain volume, or larger white matter hyperintensity volume had an increased risk of stroke compared to participants without these risk factors. The presence of multiple markers correlated with a progressively elevated risk of stroke [16]. In addition to these markers of subclinical pathology, it is hypothesized that cognitive impairment could potentially signify a clinically silent stage of a pathological progression that eventually culminates in a stroke event [45].

Finally, there are hypothesized common causes on the molecular level, as different biomarkers linked to systemic atherosclerosis and inflammatory processes, such as elevated levels of homocysteine or C-reactive protein, have been identified as being associated with risk for both cognitive impairment and stroke [46–48].

Unfortunately, both the current study and many prior studies were unable to control for these factors, and therefore were unable to determine whether the association between cognitive function and stroke risk is independent of these pathways.

### Strengths and limitations

One strength of the study is the standardized assessment of the exposure at baseline and four years later allowing us to examine cognitive performance change, an understudied risk factor for stroke. Further strengths include the adjudication of self-reported stroke events through medical record review, a low prevalence of missing data on key covariates, and the enrollment of women currently or formerly employed in health professions in the United States, which may have reduced confounding in terms of access to health care services, including diagnostic procedures.

Nevertheless, when interpreting the results, some limitations of our study should be considered.

First, we did not observe major changes in cognitive performance over four years. This may be due to the comparatively short time window during which the cognitive tests were performed, or the administered cognitive tests may not be sensitive enough to capture subtle changes in cognitive function. While the TICS may have ceiling effects, others have suggested that the effect is not as strong as those reported for the MMSE [49]. However, measurement error cannot be fully excluded, particularly due to potential ceiling and floor effects. Nevertheless, all cognitive tests were based on validated instruments and administered by trained personnel, which likely reduced measurement-related bias.

Additionally, a notable proportion of the analyzed population also demonstrated subtle improvements in cognitive performance (quintiles 5 and 4). It is possible that these improvements may capture learning or practice effects as the women completed multiple cognitive assessments with the same set of tests during the study.

A further methodological limitation in this context is regression to the mean. Given the comparatively high cognitive performance in our study sample and the possibility of measurement variability, some of the observed changes between the first and last assessment may reflect statistical artefacts rather than true changes over time.

Second, given that our study was comprised of a relatively homogeneous and health-conscious group of older, predominantly white women in health professions in the United States, the findings may not be applicable to other populations, especially to other age groups or to men, as well as populations with different risk profiles.

Third restricting the analysis to women who completed both cognitive assessments may introduce selection bias. However, a comparison of baseline characteristics and cognitive performance in the first assessment between individuals lost to follow-up and those who also participated in the final assessment did not reveal any substantial differences. Also, the results weighted for participation weights were similar to those of the primary analysis, suggesting that selection bias due to differential participation is unlikely to have substantially affected our findings.

Fourth, variables considered to result in confounding were recorded at enrollment into the main study, and we were unable to explore how time-updated covariates may mediate the effects observed.

Finally, residual and unmeasurable confounding by factors such as family history of stroke or cognitive decline, or relevant biomarkers, may exist due to the observational study design.

## Conclusion

In this large prospective cohort of United States-based women in health professions at least 65 years at baseline, we did not observe strong associations between large cognitive performance declines and stroke risk. However, further research focusing on cognitive decline rather than solely on cognitive impairment and also considering the role of cerebral and biological resilience is needed to determine if our findings are generalizable to other populations.

## Supplementary Information

Supplementary Material 1.

## Data Availability

The data underlying the analyses cannot be made publicly available as they contain information posing a risk to the confidentiality or consent of the study participants.

## Abbreviations

CI: Confidence Interval
CVD: Cardiovascular Disease
DAG: Directed Acyclic Graph after CVD
HR: Hazard Ratio
MMSE: Mini-Mental State Examination
MO: Month
OR: Odds Ratio
RR: Relative Risk
TICS: Telephone Interview of Cognitive Status
TOAST: Trial of Org 10172 in Acute Stroke Treatment
US: United States
WHS: Women’s Health Study
YR: Year
