# Supplementary material for: Cognitive decline and risk of stroke in older women: a cohort study
Source: BMC Geriatr. 2026 Jun 16;26:841. doi: 10.1186/s12877-026-07816-w (PMC13270888; doi:10.1186/s12877-026-07816-w)
Supplement: Supplementary file 1 — Supplementary Material 1. [file 12877_2026_7816_MOESM1_ESM.docx]

**Table A1**: Baseline characteristics of women lost to follow-up before last cognitive assessment and those completing it (*N* = 6,377).

|  | **Completed last assessment** (*n* = 5,226) | | **Lost to follow-up** (*n* = 1,151) | | **Overall** (*N* = 6,377) | |  |
| --- | --- | --- | --- | --- | --- | --- | --- |
| **Age** |  | |  | |  | |  |
| Mean (SD) | 66.1 (3.95) | | 66.9 (4.53) | | 66.2 (4.07) | |  |
| Median [Min, Max] | 65.4 [60.4, 87.1] | | 66.0 [60.4, 89.9] | | 65.5 [60.4, 89.9] | |  |
| **BMI** |  | |  | |  | |  |
| Mean (SD) | 25.8 (4.44) | | 25.4 (4.50) | | 25.7 (4.46) | |  |
| Median [Min, Max] | 25.1 [15.7, 51.5] | | 24.6 [14.3, 47.3] | | 25.0 [14.3, 51.5] | |  |
| Missing | 1 (0.0%) | | 4 (0.3%) | | 5 (0.1%) | |  |
| **Highest attained education** |  | |  | |  | |  |
| LPN/LVN, associate’s degree, registered nurse | 3,414 (65.3%) | | 804 (69.9%) | | 4,218 (66.1%) | |  |
| Bachelor’s degree or higher education | 1,728 (33.1%) | | 337 (29.3%) | | 2,065 (32.4%) | |  |
| Missing | 84 (1.6%) | | 10 (0.9%) | | 94 (1.5%) | |  |
| **Smoking status** |  | |  | |  | |  |
| never | 2,765 (52.9%) | | 576 (50.0%) | | 3,341 (52.4%) | |  |
| past | 1,980 (37.9%) | | 415 (36.1%) | | 2,395 (37.6%) | |  |
| current | 475 (9.1%) | | 160 (13.9%) | | 635 (10.0%) | |  |
| Missing | 6 (0.1%) | | 0 (0%) | | 6 (0.1%) | |  |
| **Strenuous physical activity** |  | |  | |  | |  |
| rarely/never | 2,220 (42.5%) | | 531 (46.1%) | | 2,751 (43.1%) | |  |
| <1 time/week | 862 (16.5%) | | 179 (15.6%) | | 1,041 (16.3%) | |  |
| 1 time per week | 432 (8.3%) | | 89 (7.7%) | | 521 (8.2%) | |  |
| 2-3 times/week | 1,093 (20.9%) | | 206 (17.9%) | | 1,299 (20.4%) | |  |
| ≥ 4 times/week | 614 (11.7%) | | 146 (12.7%) | | 760 (11.9%) | |  |
| Missing | 5 (0.1%) | | 0 (0%) | | 5 (0.1%) | |  |
| **Alcohol use** |  | |  | |  | |  |
| Rarely/never | 2,431 (46.5%) | | 608 (52.8%) | | 3,039 (47.7%) | |  |
| 1-3 drinks/month | 618 (11.8%) | | 126 (10.9%) | | 744 (11.7%) | |  |
| 1-6 drinks/week | 1,535 (29.4%) | | 277 (24.1%) | | 1,812 (28.4%) | |  |
| 1+ drinks/day | | 638 (12.2%) | | 140 (12.2%) | | 778 (12.2%) | |
| Missing | | 4 (0.1%) | | 0 (0%) | | 4 (0.1%) | |
| **Hormone replacement**  **therapy use** | |  | |  | |  | |
| never | | 2,210 (42.3%) | | 511 (44.4%) | | 2,721 (42.7%) | |
| past | | 933 (17.9%) | | 219 (19.0%) | | 1,152 (18.1%) | |
| current | | 2,076 (39.7%) | | 418 (36.3%) | | 2,494 (39.1%) | |
| Missing | | 7 (0.1%) | | 3 (0.3%) | | 10 (0.2%) | |
| **Baseline history of diabetes** | |  | |  | |  | |
| No | | 5,060 (96.8%) | | 1,093 (95.0%) | | 6,153 (96.5%) | |
| Yes | | 165 (3.2%) | | 58 (5.0%) | | 223 (3.5%) | |
| Missing | | 1 (0.0%) | | 0 (0%) | | 1 (0.0%) | |
| **Baseline history of hypertension** | |  | |  | |  | |
| No | | 3,171 (60.7%) | | 649 (56.4%) | | 3,820 (59.9%) | |
| Yes | | 2,053 (39.3%) | | 502 (43.6%) | | 2,555 (40.1%) | |
| Missing | | 2 (0.0%) | | 0 (0%) | | 2 (0.0%) | |
| **Baseline treatment of high blood pressure** | |  | |  | |  | |
| No | | 4,087 (78.2%) | | 855 (74.3%) | | 4,942 (77.5%) | |
| Yes | | 1,136 (21.7%) | | 291 (25.3%) | | 1,427 (22.4%) | |
| Missing | | 3 (0.1%) | | 5 (0.4%) | | 8 (0.1%) | |
| **Baseline history of hypercholesterolemia (cholesterol 240+)** | |  | |  | |  | |
| No | | 2,955 (56.5%) | | 680 (59.1%) | | 3,635 (57.0%) | |
| Yes | | 2,268 (43.4%) | | 471 (40.9%) | | 2,739 (43.0%) | |
| Missing | | 3 (0.1%) | | 0 (0%) | | 3 (0.0%) | |
| **Baseline treatment of hypercholesterolemia** | |  | |  | |  | |
| No | | 4,891 (93.6%) | | 1,082 (94.0%) | | 5,973 (93.7%) | |
| Yes | | 328 (6.3%) | | 69 (6.0%) | | 397 (6.2%) | |
| Missing  **BMI**: Body Mass Index (calculated as weight in kilograms divided by height in meters squared); **LPVN**: Licensed Practical Vocational Nurse | | 7 (0.1%) | | 0 (0%) | | 7 (0.1%) | |

**Table A2**: Performance of included women at first and last cognitive assessment by global cognitive performance quintiles.

|  | **1^st^ quintile** (*n* = 1,019) | **2^nd^ quintile** (*n* = 1,018) | **3^rd^ quintile** (*n* = 1,019) | **4^th^ quintile** (*n* = 1,018) | **5^th^ quintile** (*n* = 1,019) | **Overall** (*N* = 5,093) |
| --- | --- | --- | --- | --- | --- | --- |
| **TICS**^1^ |  |  |  |  |  |  |
| **First assessment** |  |  |  |  |  |  |
| Mean (SD) | 34.9 (2.48) | 34.8 (2.49) | 34.4 (2.43) | 34.3 (2.41) | 33.6 (2.57) | 34.4 (2.51) |
| Median [Min, Max] | 35.0 [23.0, 41.0] | 35.0 [25.0, 41.0] | 35.0 [24.0, 41.0] | 34.0 [23.0, 41.0] | 34.0 [21.0, 41.0] | 35.0 [21.0, 41.0] |
| **Last assessment** |  |  |  |  |  |  |
| Mean (SD) | 32.4 (3.31) | 33.8 (2.64) | 34.3 (2.56) | 35.0 (2.44) | 35.9 (2.59) | 34.3 (2.96) |
| Median [Min, Max] | 33.0 [12.0, 40.0] | 34.0 [25.0, 41.0] | 34.0 [24.0, 41.0] | 35.0 [25.0, 41.0] | 36.0 [24.0, 41.0] | 34.0 [12.0, 41.0] |
| **Immediate East Boston Memory Test**^2^ |  |  |  |  |  |  |
| **First assessment** |  |  |  |  |  |  |
| Mean (SD) | 10.2 (1.47) | 9.99 (1.42) | 9.77 (1.53) | 9.57 (1.47) | 8.79 (1.55) | 9.66 (1.56) |
| Median [Min, Max] | 10.0 [5.00, 12.0] | 10.0 [4.00, 12.0] | 10.0 [3.00, 12.0] | 10.0 [3.00, 12.0] | 9.00 [0, 12.0] | 10.0 [0, 12.0] |
| **Last assessment** |  |  |  |  |  |  |
| Mean (SD) | 8.22 (1.68) | 9.36 (1.59) | 9.84 (1.55) | 10.3 (1.52) | 10.6 (1.43) | 9.66 (1.76) |
| Median [Min, Max] | 8.00 [0, 12.0] | 10.0 [2.00, 12.0] | 10.0 [4.00, 12.0] | 10.0 [3.00, 12.0] | 11.0 [6.00, 12.0] | 10.0 [0, 12.0] |
| **Delayed East Boston Memory Test**^2^ |  |  |  |  |  |  |
| **First assessment** |  |  |  |  |  |  |
| Mean (SD) | 9.74 (1.75) | 9.67 (1.52) | 9.57 (1.55) | 9.32 (1.59) | 8.55 (1.65) | 9.37 (1.67) |
| Median [Min, Max] | 10.0 [0, 12.0] | 10.0 [0, 12.0] | 10.0 [0, 12.0] | 10.0 [0, 12.0] | 8.00 [0, 12.0] | 10.0 [0, 12.0] |
| **Last assessment** |  |  |  |  |  |  |
| Mean (SD) | 7.61 (2.43) | 9.13 (1.68) | 9.53 (1.59) | 10.0 (1.63) | 10.3 (1.47) | 9.32 (2.03) |
| Median [Min, Max] | 8.00 [0, 12.0] | 9.00 [0, 12.0] | 10.0 [0, 12.0] | 10.0 [0, 12.0] | 10.0 [0, 12.0] | 10.0 [0, 12.0] |
| **Delayed 10 word list**^3^ |  |  |  |  |  |  |
| **First assessment** |  |  |  |  |  |  |
| Mean (SD) | 3.42 (2.32) | 3.33 (2.10) | 2.99 (1.99) | 2.96 (1.84) | 2.57 (1.80) | 3.05 (2.04) |
| Median [Min, Max] | 3.00 [0, 10.0] | 3.00 [0, 10.0] | 3.00 [0, 10.0] | 3.00 [0, 10.0] | 2.00 [0, 10.0] | 3.00 [0, 10.0] |
| **Last assessment** |  |  |  |  |  |  |
| Mean (SD) | 2.26 (1.94) | 3.00 (2.03) | 3.38 (2.05) | 3.82 (2.06) | 4.77 (2.40) | 3.45 (2.26) |
| Median [Min, Max] | 2.00 [0, 10.0] | 3.00 [0, 10.0] | 3.00 [0, 10.0] | 4.00 [0, 10.0] | 4.00 [0, 10.0] | 3.00 [0, 10.0] |
| **Category fluency test**^4^ |  |  |  |  |  |  |
| **First assessment** |  |  |  |  |  |  |
| Mean (SD) | 18.4 (5.07) | 18.5 (4.96) | 17.9 (4.54) | 17.9 (4.84) | 16.6 (4.67) | 17.9 (4.87) |
| Median [Min, Max] | 18.0 [6.00, 43.0] | 18.0 [4.00, 37.0] | 18.0 [5.00, 35.0] | 18.0 [6.00, 36.0] | 16.0 [0, 33.0] | 18.0 [0, 43.0] |
| **Last assessment** |  |  |  |  |  |  |
| Mean (SD) | 15.6 (4.62) | 17.3 (4.94) | 18.1 (4.73) | 18.9 (5.11) | 19.3 (5.45) | 17.8 (5.15) |
| Median [Min, Max] | 15.0 [5.00, 32.0] | 17.0 [5.00, 38.0] | 18.0 [6.00, 44.0] | 18.0 [5.00, 38.0] | 19.0 [6.00, 49.0] | 17.0 [5.00, 49.0] |
| ***Z*-score difference [global change score]**^5^ |  |  |  |  |  |  |
| Mean (SD) | -0.929 (0.410) | -0.305 (0.114) | 0.0377 (0.0896) | 0.366 (0.107) | 0.943 (0.338) | 0.0224 (0.677) |
| Median [Min, Max] | -0.821 [-4.69, -0.512] | -0.304 [-0.512, -0.120] | 0.0370 [-0.119, 0.188] | 0.362 [0.188, 0.567] | 0.846 [0.569, 2.76] | 0.0370 [-4.69, 2.76] |

^1^ Telephone Interview of Cognitive Status (TICS) (max. 41 points)

^2^ Max. 12 points

^3^ Max. 10 points

^4^ Women were asked to name as many animals as possible in one minute

^5^ Global test scores were generated by averaging Z-scores of the performance in each of the five tests

**Table A3**: Performance of eligible women at first cognitive assessment by follow-up status (*N* = 6,377).

| **First assessment** | **Completed last assessment** (*n* = 5,226) | **Lost to follow-up** (*n* = 1,151) | **Overall** (*N* = 6,377) |
| --- | --- | --- | --- |
| **TICS**^1^ |  |  |  |
| Mean (SD) | 34.4 (2.54) | 33.5 (3.39) | 34.2 (2.73) |
| Median [Min, Max] | 35.0 [21.0, 41.0] | 34.0 [4.00, 41.0] | 34.0 [4.00, 41.0] |
| Missing | 0 (0%) | 15 (1.3%) | 15 (0.2%) |
| **Immediate East Boston Memory Test**^2^ |  |  |  |
| Mean (SD) | 9.65 (1.56) | 9.25 (1.68) | 9.58 (1.59) |
| Median [Min, Max] | 10.0 [0, 12.0] | 10.0 [0, 12.0] | 10.0 [0, 12.0] |
| Missing | 1 (0.0%) | 4 (0.3%) | 5 (0.1%) |
| **Delayed East Boston Memory Test^2^** |  |  |  |
| Mean (SD) | 9.36 (1.67) | 8.90 (2.20) | 9.28 (1.79) |
| Median [Min, Max] | 10.0 [0, 12.0] | 9.00 [0, 12.0] | 10.0 [0, 12.0] |
| Missing | 0 (0%) | 16 (1.4%) | 16 (0.3%) |
| **Immediate 10 word list**^3^ |  |  |  |
| Mean (SD) | 4.87 (1.73) | 4.63 (1.92) | 4.83 (1.77) |
| Median [Min, Max] | 5.00 [0, 10.0] | 4.00 [0, 10.0] | 5.00 [0, 10.0] |
| Missing | 1 (0.0%) | 12 (1.0%) | 13 (0.2%) |
| **Delayed 10 word list**^3^ |  |  |  |
| Mean (SD) | 3.04 (2.04) | 2.71 (2.33) | 2.98 (2.10) |
| Median [Min, Max] | 3.00 [0, 10.0] | 2.00 [0, 10.0] | 3.00 [0, 10.0] |
| Missing | 0 (0%) | 16 (1.4%) | 16 (0.3%) |
| **Category fluency test^4^** |  |  |  |
| Mean (SD) | 17.8 (4.87) | 16.1 (5.07) | 17.5 (4.95) |
| Median [Min, Max] | 18.0 [0, 43.0] | 16.0 [0, 34.0] | 17.0 [0, 43.0] |
| Missing | 1 (0.0%) | 23 (2.0%) | 24 (0.4%) |

^1^ Telephone Interview of Cognitive Status (TICS) (max. 41 points)

^2^ Max. 12 points

^3^ Max. 10 points

^4^ Women were asked to name as many animals as possible in one minute

**Table A4**:  HRs for total stroke by global cognition, weighted by participation weights

| **Global cognitive performance quintiles:** | 1^st^ quintile (*n* = 1,019)  HR (95%CIs) | 2^nd^ quintile (*n* = 1,018)  HR (95%CIs) | 3^rd^ quintile (*n* = 1,019)  HR (95%CIs) | 4^th^ quintile (*n* = 1,018)  HR (95%CIs) | 5^th^ quintile (*n* = 1,019)  HR [reference] |
| --- | --- | --- | --- | --- | --- |
| **Stroke, *N*** | 60 | 62 | 60 | 53 | 67 |
| adjusted^1^ | 1.08 (0.76-1.54) | 1.02 (0.71-1.45) | 0.99 (0.69-1.41) | 0.79 (0.54-1.15) | 1 (ref) |

Abbreviations: CI: Confidence Interval, HR: Hazard Ratio, ref: reference group

^1^Adjusted for age, strenuous physical activity, alcohol use, smoking status, BMI (calculated as weight in kilograms divided by height in meters squared), highest attained educational level, hormone replacement therapy, high blood pressure, treatment of high blood pressure, high cholesterol, treatment of high cholesterol, and diabetes at baseline

**
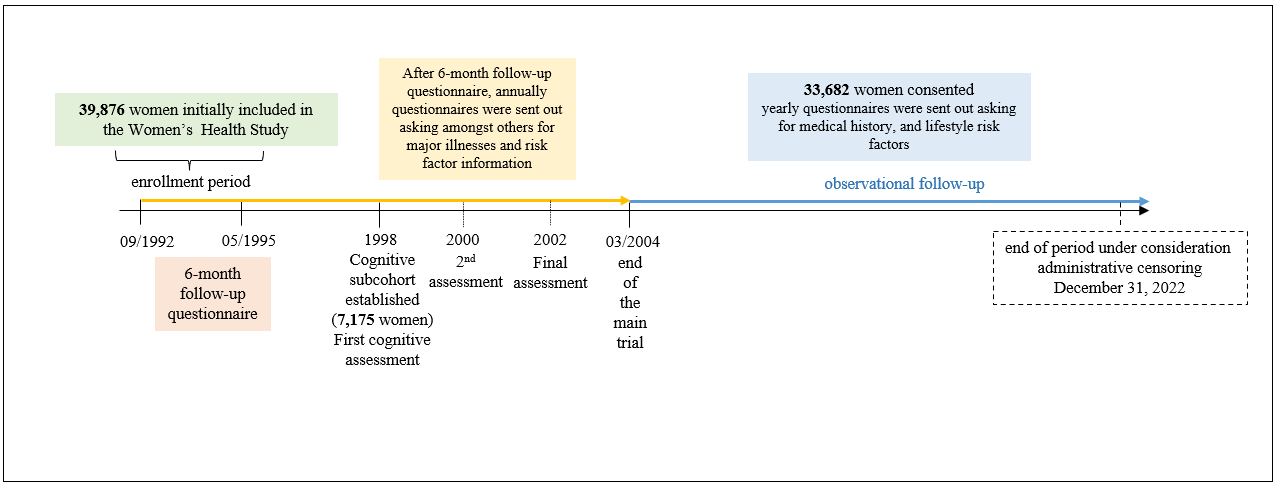
**

**eFigure1**: Timeline plot of the Women’s Health Study, the cognitive subcohort, and of the data collection procedure.

**
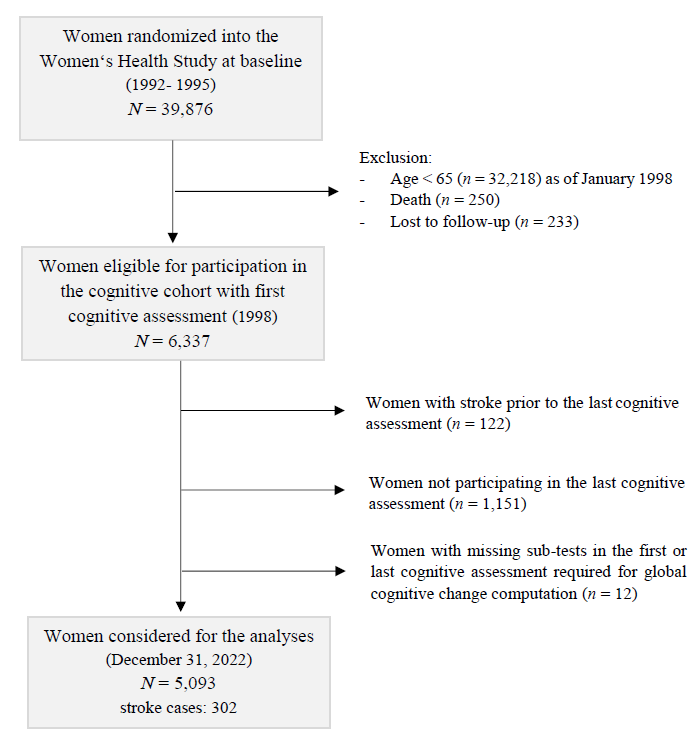
**

**eFigure 2**: Flow chart for participant selection and exclusions from the WHS (cognitive cohort).


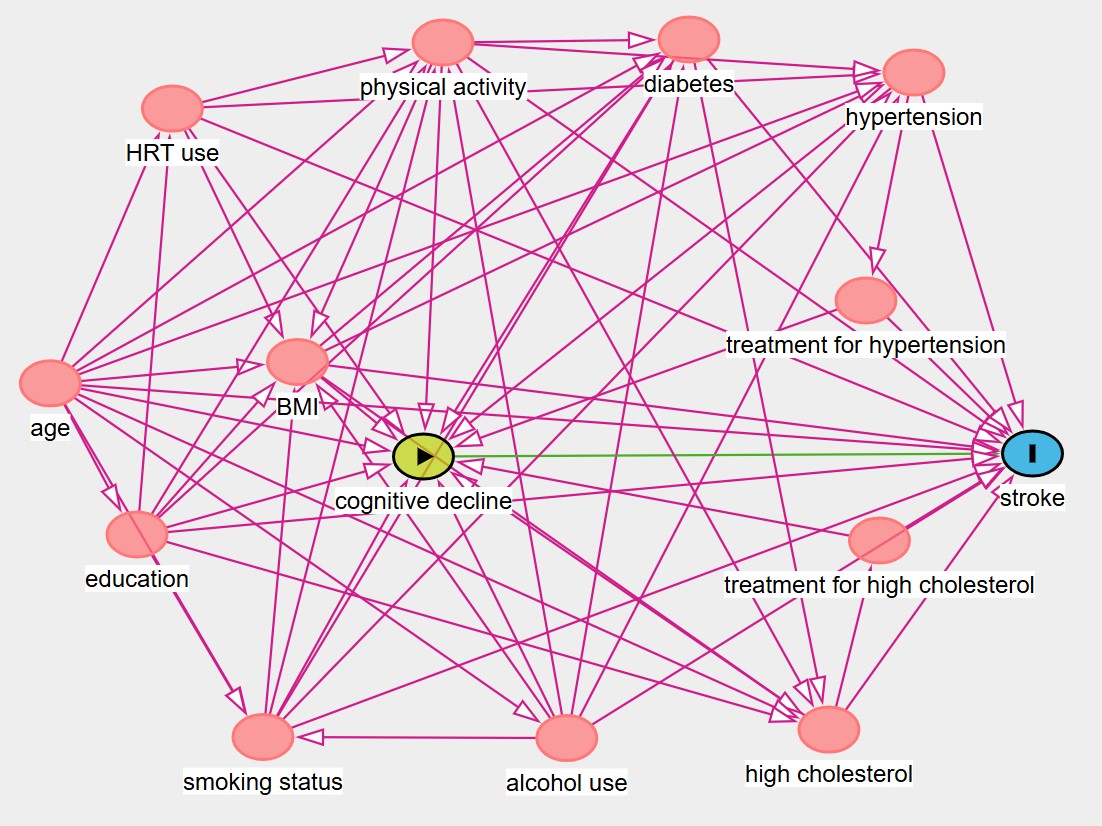


BMI: Calculated as weight in kilograms divided by height in meters squared

HRT: Hormone replacement therap**y**

**eFigure 3**: Directed acyclic graph (DAG) for the effect of *“cognitive decline“* on *“stroke”.*

*
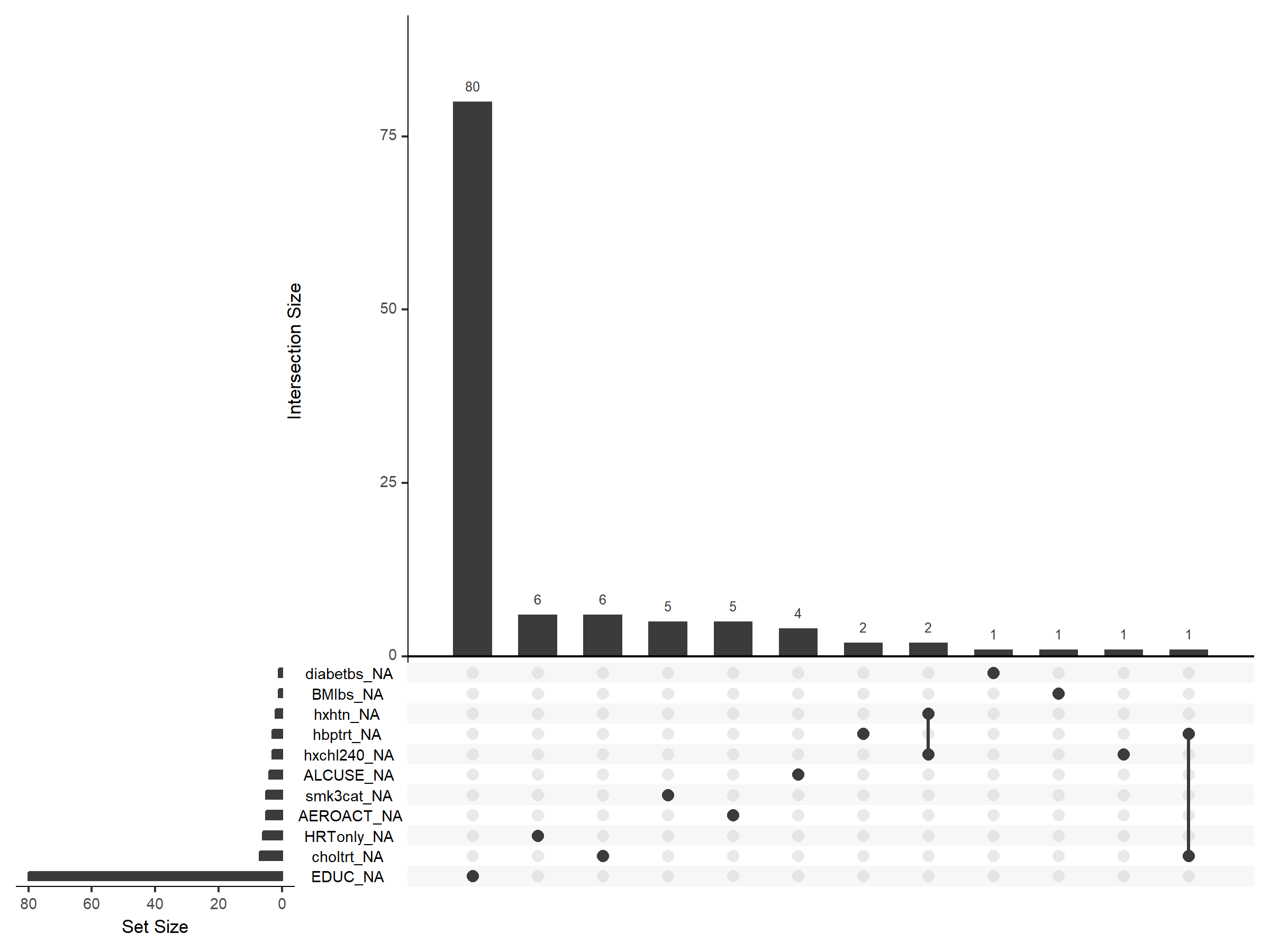
*

diabetbs: diabetes at baseline; BMIbs: Body Mass Index at baseline; hxhtn: hypertension; hbptrt: treatment for high blood pressure; hxchl240: high cholesterol; ALCUSE: alcohol use category; smk3cat: smoking status (three categories); AEROACT: physical activity level; HRTonly: Hormone Replacement Therapy; choltrt: treatment for high cholesterol; EDUC: educational level

**eFigure4**: UpSet plot showing numbers and patterns of missing data across adjustment variables.


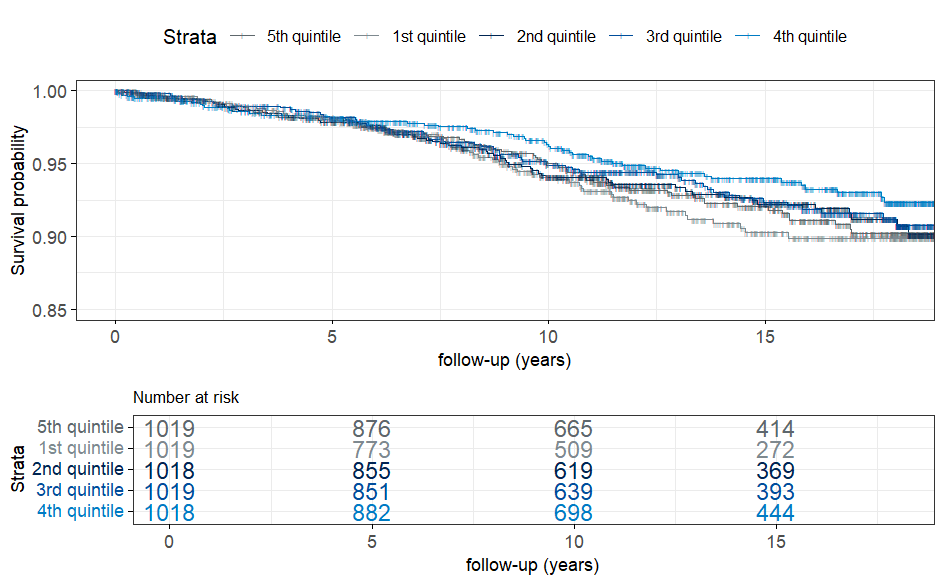


Person-time was calculated from the last cognitive assessment until the date of diagnosis of confirmed stroke, loss to follow-up, death, or the end of the study (December 31, 2022), whichever occurred first.

**eFigure 5**: Kaplan Meier curves for stroke-free survival of included women from the WHS cognitive cohort (*N* = 5,093).
